# A phase 2 trial investigating the efficacy and safety of the mPGES-1 inhibitor vipoglanstat in systemic sclerosis-related Raynaud's

**DOI:** 10.1093/rheumatology/keae049

**Published:** 2024-01-30

**Authors:** Göran Tornling, Charlotte Edenius, John D Pauling, Christopher P Denton, Anna Olsson, Jan Kowalski, Andrea Murray, Marina Anderson, Smita Bhat, Francesco Del Galdo, Frances Hall, Mariusz Korkosz, Dorota Krasowska, Jacek Olas, Vanessa Smith, Jacob M van Laar, Madelon C Vonk, Anna Wojteczek, Ariane L Herrick

**Affiliations:** Respiratory Medicine Division, Department of Medicine Solna, Karolinska Institutet, Stockholm, Sweden; Gesynta Pharma AB, Stockholm, Sweden; Gesynta Pharma AB, Stockholm, Sweden; Rheumatology Division, Department of Medicine Solna, Karolinska Institutet, Stockholm, Sweden; Department of Rheumatology, North Bristol NHS Trust, Bristol, UK; Musculoskeletal Research Unit, Translational Health Sciences, Bristol Medical School, University of Bristol, Bristol, UK; Royal National Hospital for Rheumatic Diseases (part of the Royal United Hospitals NHS Foundation Trust), Bath, UK; Centre for Rheumatology, Royal Free Hospital and University College London, London, UK; Gesynta Pharma AB, Stockholm, Sweden; JK Biostatistics AB, Stockholm, Sweden; Centre for Musculoskeletal Research, The University of Manchester, Northern Care Alliance NHS Foundation Trust, Manchester Academic Health Science Centre, Manchester, UK; Aintree University Hospital, Liverpool University Hospitals NHS Trust, Liverpool, UK; Lancaster Medical School, Lancaster University, Lancaster, UK; Ninewells Hospital and Medical School, Dundee, UK; Leeds Institute of Rheumatology & Musculoskeletal Medicine, and NIHR Leeds Biomedical Research Centre, University of Leeds, Leeds, UK; Cambridge University Hospitals NHS Foundation Trust, Cambridge, UK; Centrum Medyczne Pratia MCM Krakow, Krakow, Poland; Jagiellonian University Medical College, Krakow, Poland; Department of Dermatology, Venereology and Pediatric Dermatology Medical University of Lublin, Poland; Małopolskie Centrum Kliniczne, Kraków, Poland; Department of Internal Medicine, Ghent University, Ghent, Belgium; Department of Rheumatology, Ghent University Hospital, Ghent, Belgium; Unit for Molecular Immunology and Inflammation, VIB Inflammation Research Center (IRC), Ghent, Belgium; University Medical Center Utrecht, Utrecht, The Netherlands; Department of Rheumatology, Radboud University Medical Center, Nijmegen, The Netherlands; Early Phase Clinical Trials Centre, Medical University of Gdańsk, Gdańsk, Poland; Department of Rheumatology, Clinical Immunology, Geriatrics and Internal Medicine, Medical University of Gdańsk, Gdańsk, Poland; Centre for Musculoskeletal Research, The University of Manchester, Northern Care Alliance NHS Foundation Trust, Manchester Academic Health Science Centre, Manchester, UK

**Keywords:** clinical trial, microsomal prostaglandin E synthase-1, vipoglanstat, Raynaud’s phenomenon, systemic sclerosis

## Abstract

**Objective:**

Our objective was to test the hypothesis, in a double-blind, placebo-controlled study that vipoglanstat, an inhibitor of microsomal prostaglandin E synthase-1 (mPGES-1), which decreases prostaglandin E_2_ (PGE_2_) and increases prostacyclin biosynthesis, improves RP.

**Methods:**

Patients with SSc and ≥7 RP attacks during the last screening week prior to a baseline visit were randomized to 4 weeks treatment with vipoglanstat 120 mg or placebo. A daily electronic diary captured RP attacks (duration and pain) and Raynaud’s Condition Score, with change in RP attacks/week as the primary end point. Cold challenge assessments were performed at baseline and end of treatment. Exploratory end points included patients’ and physicians’ global impression of change, Assessment of Scleroderma-associated Raynaud’s Phenomenon questionnaire, mPGES-1 activity, and urinary excretion of arachidonic acid metabolites.

**Results:**

Sixty-nine subjects received vipoglanstat (*n* = 33) or placebo (*n* = 36). The mean weekly number of RP attacks [baseline; vipoglanstat 14.4 (S.D. 6.7), placebo 18.2 (12.6)] decreased by 3.4 (95% CI –5.8; –1.0) and 4.2 (–6.5; –2.0) attacks per week (*P* = 0.628), respectively. All patient-reported outcomes improved, with no difference between the groups. The mean change in recovery of peripheral blood flow after the cold challenge did not differ between the study groups. Vipoglanstat fully inhibited mPGES-1, resulting in 57% reduction of PGE_2_ and 50% increase of prostacyclin metabolites in the urine. Vipoglanstat was safe and well tolerated.

**Conclusion:**

Although vipoglanstat was safe, and well tolerated in a dose achieving full inhibition of mPGES-1, it was ineffective in SSc-related RP. Further development and evaluation of vipoglanstat will therefore be in other diseases where mPGES-1 plays a pathogenetic role.

**Trial registration:**

ClinicalTrials.gov, https://www.clinicaltrials.gov, NCT0474420.

Rheumatology key messages

- Vipoglanstat inhibits microsomal prostaglandin E synthase-1 (mPGES-1), decreasing prostaglandin E_2_ (PGE_2_) and increasing prostacyclin biosynthesis.
- A randomized, double-blind, placebo-controlled parallel group study of vipoglanstat in SSc-related Raynaud’s phenomenon is reported.
- Despite full mPGES-1 inhibition, treatment with vipoglanstat did not improve Raynaud’s or finger blood flow.

## Introduction

Almost all patients with SSc experience RP [1], episodic colour change of the extremities (most noticeably the fingers) in response to cold exposure or emotional stress. Attacks of RP are often painful and disabling, and SSc-related RP has a major impact on quality of life: the results of a survey of almost 2000 patients with SSc suggested that RP was the ‘organ’ complication that had the most impact on daily life [2]. Current treatments are only partially effective (if at all). Therefore, new, effective therapies are required.

One way to improve blood supply to the fingers in patients with SSc is via supplementation of the prostacyclin pathway, as in the treatment of pulmonary arterial hypertension [3]. I.v. iloprost has been shown to reduce frequency and severity of RP attacks in patients with SSc [4, 5], and to improve temperature recovery to the fingers after a cold challenge [6], but has the disadvantage of requiring hospitalization. Oral therapies would be advantageous. However, studies of oral prostanoid therapy have been disappointing [4, 5]. The most recent randomized controlled trial of oral prostanoid therapy in SSc was primarily a study of digital ulceration, with limited assessment of RP [7]: treprostinil conferred no benefit compared with placebo as measured by a visual analogue scale (VAS) for interference of RP with daily activities, although there was some RP benefit in terms of the ‘patient assessment of change’ questionnaire. The prostacyclin receptor agonist selexipag conferred no benefit in a placebo-controlled trial in patients with SSc-related RP [8].

**Figure 1.**
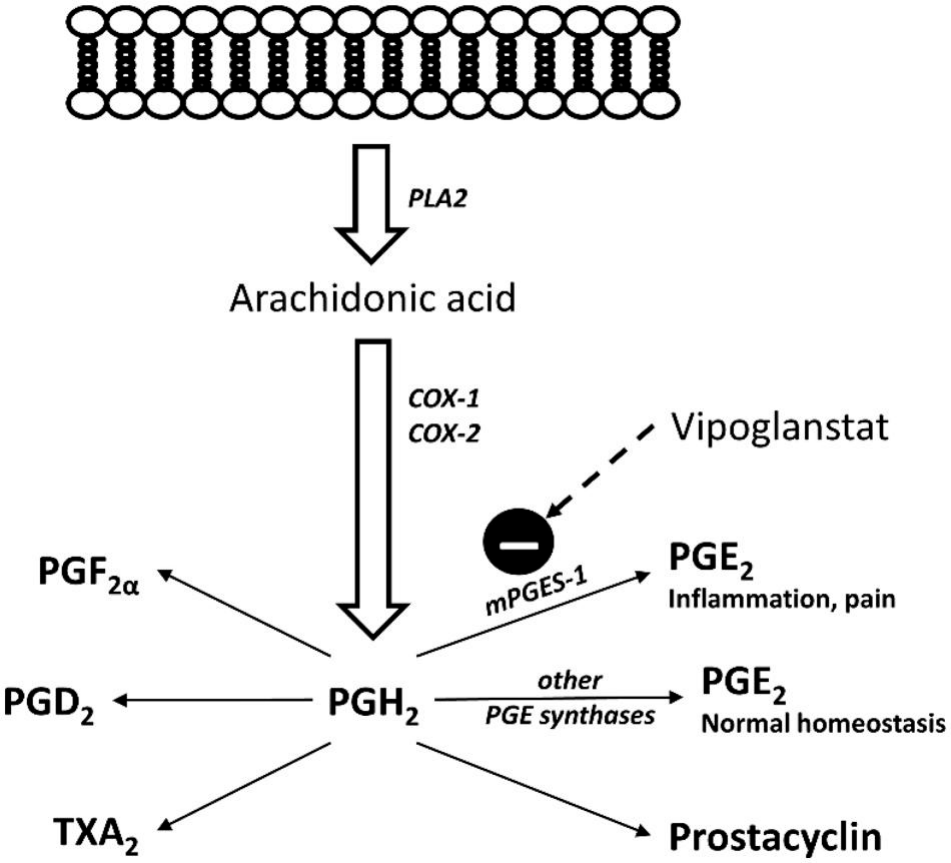
Arachidonic acid metabolism. Arachidonic acid, released from the cell membrane by phospholipase A2 (PLA2), is by cyclooxygenases (COX-1 and COX-2) metabolized to the unstable PGH_2_, which by different terminal synthases is converted to different prostaglandins. The inducible enzyme prostaglandin E_2_ synthase-1 (mPGES-1) is associated with inflammation and pain, while other PGE synthases maintain normal homeostasis. PLA2: phospholipase A2; PGE_2_: prostaglandin E_2_; PGI_2_: prostacyclin; TXA_2_: thromboxane A_2_; PGD_2_: prostaglandin D_2_; PGF_2α_: prostaglandin F_2_alpha; PGH_2_: prostaglandin H_2_

A novel way of augmenting the prostacyclin pathway is through inhibition of microsomal prostaglandin E_2_ synthase-1 (mPGES-1), which is a terminal enzyme converting the unstable cyclooxygenase-derived PGH_2_ to prostaglandin E_2_ (PGE_2_). Other synthases convert PGH_2_ to prostacyclin, thromboxane and other metabolites in the arachidonic acid cascade (Fig. 1). By inhibition of mPGES-1, PGH_2_ is shunted towards other metabolites, and increased prostacyclin synthesis has been demonstrated in mouse peritoneal macrophages [9] and mPGES-1 knock-out mice [10, 11], as well as by pharmacological inhibition of mPGES-1 in humans [12, 13]. Shunting of PGH_2_ towards biosynthesis of prostacyclin might be beneficial in patients with SSc-related RP.

Vipoglanstat (also known as GS-248 and BI 1029539) is a selective inhibitor of mPGES-1. The compound is highly potent, with IC_50_ in *ex vivo* human whole blood estimated to be ≤0.5 nmol/l in a phase 1 study in healthy volunteers [12]; vipoglanstat at doses fully inhibiting mPGES-1 was safe and well tolerated, and urinary analyses showed decreased levels of PGE_2_ and increased levels of prostacyclin metabolites.

This study aimed to test the hypothesis that, in patients with SSc, treatment with vipoglanstat increases endogenous production of prostacyclin and decreases the RP attack frequency and severity. Other specific objectives were to evaluate the safety and efficacy of vipoglanstat on peripheral blood flow in response to cold challenge, inhibition of mPGES-1 activity and excretion of arachidonic acid metabolites in urine. The correlation between change in Assessment of Scleroderma-associated Raynaud’s Phenomenon (ASRAP) questionnaire score and patient global impression of change was explored in a *post hoc* analysis.

## Material and methods

### Study design

This was a Phase 2, randomized, double-blind, placebo-controlled, parallel group study (ClinicalTrials.gov, NCT04744207) with a 1:1 allocation of participants to either three capsules of 40 mg (total dose 120 mg) vipoglanstat, or corresponding identical placebo capsules, for oral intake once daily in the morning for 4 weeks. The blinding was maintained throughout the study. Patients were enrolled by the investigator at each study site. Patients were scheduled to have five visits: initial screening (2–3 weeks before randomization), randomization and start of treatment (baseline visit), safety assessments (2 weeks after randomization), end of treatment (4 weeks after randomization), and an end-of-study visit (2–3 weeks after last treatment) (Supplementary Fig. S1, available at *Rheumatology* online). The allocation codes were masked and not broken until declaration of a clean file, which kept patients, investigators and outcome assessors blinded throughout the study. The study was approved by appropriate independent ethics committees, and informed consent was obtained from all patients.

### Eligibility criteria

Male and female non-smoking patients were eligible for recruitment if they fulfilled the EULAR/ACR 2013 criteria for SSc [14], with a disease duration of ≤120 months from first non-Raynaud’s manifestation, were aged 18–75 years and had a typical frequency of RP attacks of ≥7 times per week during the 4 weeks prior to screening, despite background medication [calcium channel blockers or phosphodiesterase type 5 (PDE-5) inhibitors]. To be eligible for randomization, patients were required to have ≥7 RP attacks during the last week of the run-in period, with no >2 days without RP attacks. During the 4 weeks prior to screening, patients should not have changed dose or initiated treatment with vasodilating substances. The use of calcium channel blockers or PDE-5 inhibitors in a stable dose was allowed throughout the study. Further, the patients should not have been treated with prostacyclin receptor agonists, systemic CSs or immunosuppressive therapy other than stable doses of MMF, and they should not have had an active digital ulcer within 4 weeks prior to screening. The full eligibility criteria are presented in the Supplementary Material, available at *Rheumatology* online.

### Primary and key secondary end points

From screening to end of treatment, the patients completed a daily electronic diary, including number of Raynaud’s attacks, the length (min) and pain [Numerical Rating Scale (NRS) 0–10] of each RP attack, and daily assessment of the Raynaud’s Condition Score (RCS) (NRS 0–10).

The primary end point was defined as the mean change in number of Raynaud’s attacks from the last 7 days of screening to the last 7 days of treatment, as assessed by the electronic diary. The diary also included assessments for the key secondary end points, which were defined as: mean change from baseline to week 4 in (i) pain experience during RP attacks, (ii) RCS Score, (iii) cumulative duration of RP attacks (log-transformed), and (iv) mean duration of RP attacks (log-transformed).

### Thermography and cold challenge

Thermographic end points at Visit 2 and Visit 4 were evaluated only at selected sites, based on the researcher’s experience in the methodology. All measurements were performed in a temperature-controlled (23 ± 2°C) or temperature-monitored room. Patients were requested to wear light clothing and refrain from vigorous exercise, caffeine and alcohol for at least 4 h prior to the assessment. Upon arrival, patients were seated comfortably in the temperature-controlled (or temperature-monitored) room for 20 min and acclimatized. At Visit 2 assessments were performed before and 180 min after study drug administration, and at Visit 4 immediately before study drug administration.

The temperature of the dorsum of each hand and all eight distal phalanges (excluding thumbs) was measured. The distal dorsal difference (DDD), defined as the difference in measurements between the dorsum and distal phalanx, was calculated for each finger. For comparison between the treatment groups, the mean finger temperature and DDD for all eight fingers was calculated.

The cold challenge involved a 1 min exposure to 15°C water for both hands to the MCP joints and rewarming at ambient temperature. Immediately prior to the cold challenge, a baseline image of both hands was taken with the thermal camera, with the subject’s hands placed on a thermally insulated surface. Rewarming of the fingers after the cold challenge was imaged with 4 frames per min over 15 min following hand removal from the water, and the averaged areas under the curve (AUC) and maximum (MAX) values for all eight fingers were calculated [15].

The mean change from Visit 2 (pre study drug assessments) to Visit 4 were predefined as secondary end points, and the change between the two assessments at Visit 2 as exploratory end points.

### mPGES-1 activity and urinary excretion of arachidonic acid metabolites

Inhibition of the target enzyme (mPGES-1) was assessed on blood samples drawn prior to study drug administration on Visit 2 and Visit 4, and analysed by a qualified whole-blood assay, with the results expressed as percentage change. Quantification of the urinary excretion of metabolites derived from PGE_2_ (PGEM), prostacyclin (PGIM) and thromboxane (TXM) was performed on the morning urine at Visit 2 and Visit 4, with the results expressed as percentage change after normalization to urinary creatinine (for details on the methods, see the supplementary methods, available at *Rheumatology* online).

### Other exploratory end points

Other exploratory end points included patients’ and physicians’ global impression of change (PaGIC and PhGIC) assessed at end of treatment, and the mean change from baseline to end of treatment in the ASRAP questionnaire total score [16, 17]. The ASRAP questionnaire was assessed at baseline (Visit 2) and all subsequent visits using the preliminary 39-item beta version, and scores were *post hoc* calculated using the algorithm for the final 27-item version [16, 17].

### Nailfold capillaroscopy

At baseline, patients underwent nailfold capillaroscopy, and the investigator classified the pattern as normal/early/active/late [18], with the results expressed as either the ‘majority’ verdict across all fingers, or in the case of a tie the worst grade. Frames (usually four per finger) were quantitatively assessed automatically regarding capillary density and width (after manual identification of the location of the capillary apices) as previously described [19]. Giant capillaries were defined as present if the maximal capillary width was >50 μm in any finger.

### Safety assessments

Evaluation of safety was a pre-defined secondary objective. All adverse events, serious and non-serious, reported from the first day of study treatment up until and including 7 days after the last dose of the study treatment were considered treatment-emergent adverse events (AEs). At all study visits haematological, blood chemistry and urine analyses were performed. ECGs were recorded at inclusion, randomization, 2 weeks after start of treatment, and at the follow-up visit. An additional ECG was recorded at the assumed peak exposure 170 min after the first administration of the study drug.

### Statistics

Sample-size calculation was done for the primary end point and based on a two-sided *t* test for independent samples with a type-I error rate of 0.05. Calculations assumed a mean change from baseline to week 4 of 12.0 attacks per week for the active treatment arm *vs* 4.0 for the placebo arm, and a common S.D. of 10.0, with a power of 80%. The calculations revealed that 26 patients per group were needed; to allow for dropouts, it was planned to randomize ∼80 patients into the study.

The random allocation sequence in blocks of four was generated by an independent statistician not involved in any other aspects of the study. Randomization was performed at a central site, with stratification based on background vasodilatory treatment (calcium channel blockers, PDE-5 inhibitors, or no background vasodilatory treatment).

The statistical analyses for the primary end point and all other continuous end points were performed using analysis of covariance (ANCOVA), including the stratification factor and treatment as fixed factors, and baseline levels as a covariate. Analysis was done for the full analysis (FAS) population, which consisted of all patients who were randomized and received at least one dose of investigational medicinal product (IMP). For the primary end point, missing data at week 4 was replaced using the placebo multiple imputation (pMI) method, i.e. imputing the placebo behaviour for missing data, utilizing multiple draws from the posterior predictive distribution estimated from the placebo arm. There was no imputation for missing data in other end points. Due to the skew distribution of Raynaud’s attack duration (single attacks and cumulatively over a week) and temperature after cold challenge, these variables were analysed on log-transformed (natural logarithm) values. Categorical end points such as PaGIC and PhGIC at week 4 were analysed using the Cochran Mantel–Haenzel method, adjusted for the stratification factor. The change from baseline to end of treatment in mPGES-1 activity assessed by PGE_2_ formation in the whole blood assay and urinary excretion of arachidonic acid metabolites was analysed by the Mann–Whitney *U* test.

The overall type I family-wise error rate for testing the primary and the key secondary efficacy end points was controlled at the type-I error rate of 0.05 using a serial gatekeeping multiple comparisons procedure. Following this multiple comparisons procedure, progression to the next end point only occurred if the null hypothesis was rejected. Since the null hypothesis was not rejected for the primary efficacy end point, all subsequent hypothesis tests were rejected, and the reported *P*-values should be regarded as nominal.

In a *post hoc* analysis, correlation between PaGIC and change in ASRAP score from baseline to end of treatment was estimated using the Spearman rank order correlation coefficient.

## Results

**Figure 2.**
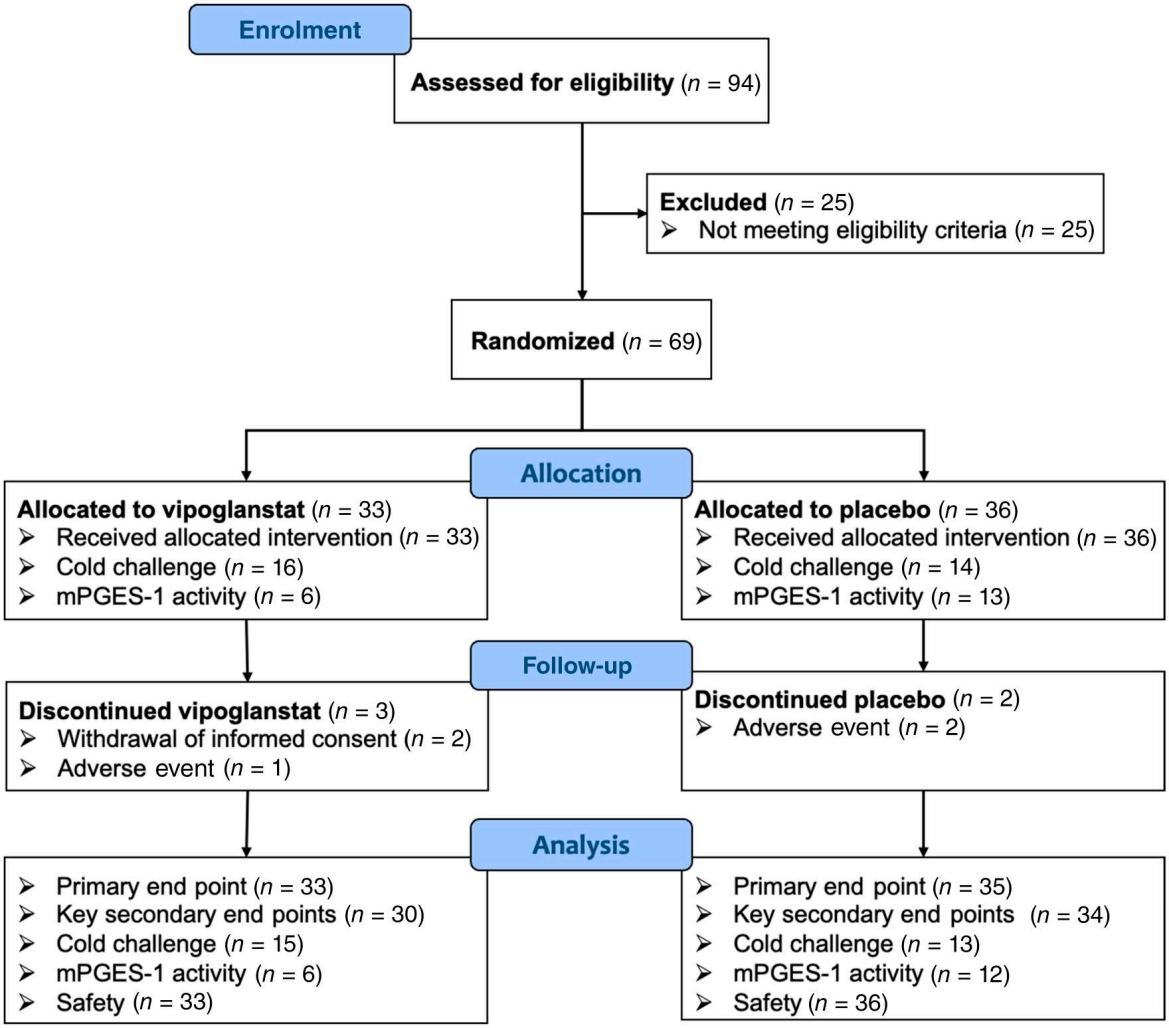
Patient disposition. Primary and key secondary end points were analysed for the FAS population, with missing data at week 4 for the primary end point replaced using the placebo multiple imputation (pMI) method. For one subject in the placebo group, the screening period was extended due to an intercurrent medical event, and the electronic diary could not capture a RP attack during the last week of the run-in period. Since this subject had fulfilled the screening criteria earlier during the run-in period, the subject was considered as eligible and was randomized into the study, but as data on RP attacks were not captured during the defined baseline period, the subject was excluded from the end point analyses on RP attacks. FAS: full analysis; mPGES-1: prostaglandin E synthase-1

The study was conducted from 29 December 2020 (first patient first visit) to 15 June 2022 (last patient last visit) at 14 sites in four European countries. No patients were enrolled between 20 April and 22 September 2021 to minimize the effect of warmer weather on the efficacy outcome variables. Recruitment was permanently halted when 69 patients were randomized, since the warmer summer months were approaching, and it was assumed, based on actual drop-out rate, that at least 26 patients had been allocated to each treatment arm as stipulated in the power calculations. The study over-ran by one winter season, mainly due to recruitment challenges during the COVID-19 pandemic. A total of 94 patients were enrolled for screening, of whom 25 failed the eligibility criteria. Out of the 69 randomized patients, 33 were allocated to vipoglanstat and 36 to placebo (Fig. 2). At pre-selected sites, patients were assessed by thermography, cold challenge, and whole-blood assay for mPGES-1 activity. Three patients in the vipoglanstat group and two patients in the placebo group withdrew during the treatment period. The baseline characteristics were comparable between the two groups (Table 1).

**Table 1.** Patient characteristics

	Treatment group	
	Vipoglanstat (*N* = 33)	Placebo (*N* = 36)
Females	27 [81.8%]	33 [91.7%]
Race		
− Asian	1 [3.0%]	0 [0.0%]
− White	30 [90.9%]	34 [94.4%]
− Not collected	2 [6.1%]	2 [5.6%]
Age (years)	49.0 (10.6)	50.6 (10.6)
Weight (kg)	74.5 (16.4)	68.9 (14.1)
BMI (kg/m²)	26.1 (5.0)	25.1 (4.4)
Duration of RP (months)^a^	122.1 (135.1)	93.1 (68.6)
Duration of SSc from first non-RP manifestation (months)^b^	37.4 (33.5)	52.0 (32.4)
Capillaroscopy pattern^c^		
− Normal	3 [9.1%]	3 [8.3%]
− Early	9 [27.3%]	9 [25.0%]
− Active	11 [33.3%]	11 [30.6%]
− Late	10 [30.3%]	13 [36.1%]
Capillary dimensions^d^		
− Capillary density (per mm); mean 8 fingers	5.9 (2.3)	5.9 (1.8)
− Mean width (µm); mean 8 fingers	31.1 (7.5)	29.8 (6.3)
− Presence of giant capillaries	25 [86.2%]	26 [83.9%]
Autoantibodies		
− ANA positive	33 [100.0%]	35 [97.2%]
− ACA positive	16 [48.5%]	20 [55.6%]
− Anti-Scl-70 antibody positive	13 [39.4%]	12 [33.3%]
Background vasodilatory treatment		
− Calcium channel blockers	18 [54.6%]	19 [52.8%]
− PDE-5 inhibitors	9 [27.3%]	10 [27.8%]
− No vasodilatory treatment	6 [18.2%]	7 [19.4%]

Values are number [percentage] for categorical variables and mean (S.D.) for continuous variables.

a Placebo *N* = 33.

b Vipoglanstat *N* = 32, placebo *N* = 31.

c Majority verdict of eight fingers, or in the case of a tie the worst grade.

d Vipoglanstat *N* = 29, placebo *N* = 31.

### Primary and key secondary end points (RP attacks and RCS)

There was no statistically significant difference between the vipoglanstat and placebo groups regarding the primary end point, mean change from baseline to week 4 in the number of RP attacks per week (*P* = 0.628). The mean change in number of attacks decreased in both the vipoglanstat and placebo groups by 3.4 and 4.2 attacks per week, respectively. Further, there were no statistically significant differences between the vipoglanstat and placebo groups on the key secondary end points (cumulative duration of and pain experienced during attacks, and RCS), which all improved in both the vipoglanstat- and placebo-treated groups (Table 2). The results were not influenced by use of background vasodilatory treatment (data not shown).

**Table 2.** Change from baseline to end of treatment in primary, secondary and exploratory end points

	Baseline (BL)		End of treatment (EoT)		**LS mean change from baseline to end of treatment** ^a^			
	Vipoglanstat	Placebo	Vipoglanstat	Placebo	Vipoglanstat	Placebo	Vipoglanstat – Placebo	*P* value
**Electronic diary**	*N = 33*	*N = 35*	*N = 30*	*N = 34*				
Number of attacks per week	14.4 (6.7)	18.2 (12.6)	11.1 (9.5)	14.0 (12.8)	–3.41 [–5.83; –0.99]	–4.22 [–6.48; –1.96]	0.81 [–2.48: 4.10]	0.628
Cumulative duration of attacks (min)	525.5 (460.4)	480.6 (479.6)	450.4 (571.8)	356.0 (320.6)				
ln(cumulative duration of attacks)^b^	5.9 (0.82)	5.8 (0.81)	5.6 (1.04)	5.4 (1.20)	0.70 [0.51; 0.98]	0.61 [0.45; 0.82]	1.16 [0.75; 1.81]	0.506
Mean attack duration (min)	38.5 (36.0)	27.4 (19.2)	43.4 (51.5)	25.7 (14.6)				
ln(mean attack duration)^b^	3.4 (0.72)	3.1 (0.65)	3.4 (0.82)	3.1 (0.66)	1.06 [0.87; 1.29]	0.89 [0.74; 1.08]	1.18 [0.90; 1.56]	0.231
Mean pain during attack (NRS 0–10)	3.8 (2.0)	4.0 (2.1)	3.0 (2.0)	3.4 (2.3)	–0.65 [–1.16; –0.14]	–0.60 [–1.07; –0.13]	–0.05 [–0.74; 0.64]	0.891
RCS (NRS 0–10)	4.1 (2.2)	4.1 (2.1)	2.9 (2.2)	3.2 (2.2)	–0.99 [–1.54; –0.45]	–0.95 [–1.45; –0.46]	–0.04 [–0.78; 0.70]	0.918
**Hand and finger temperature**	*N = 16*	*N = 14*	*N = 12*	*N = 13*				
DDD (°C)	–1.58 (1.16)	–2.13 (1.89)	–1.53 (1.81)	–2.43 (1.85)	0.27 [–0.87; 1.40]	–0.05 [–1.19; 1.08]	0.32 [–1.23; 1.87]	0.671
Mean finger temperature (°C)	28.51 (2.56)	28.27 (3.44)	28.18 (3.07)	27.77 (3.28)	–0.15 [–2.22; 1.93]	–0.22 [–2.28; 1.85]	0.07 [–2.71; 2.85]	0.958
**Finger temperature after cold challenge**	*N = 16*	*N = 15*	*N = 14*	*N = 13*				
AUC (°C*sec)	22 670 (2961)	22 496 (2916)	23 083 (2008)	22 999 (3294)				
ln(AUC)^b^	10.02 (0.126)	10.01 (0.121)	10.04 (0.085)	10.03 (0.136)	1.05 [0.98; 1.13]	1.04 [0.97; 1.11]	1.01 [0.92; 1.11]	0.825
Max temperature (°C)	26.8 (4.17)	26.5 (4.03)	27.7 (3.61)	26.8 (4.36)				
ln(Max temperature)^b^	3.28 (0.151)	3.27 (0.143)	3.31 (0.129)	3.28 (0.154)	1.07 [0.97; 1.17]	1.03 [0.94; 1.13]	1.04 [0.92; 1.17]	0.559
**Exploratory end point**	*N = 33*	*N = 36*	*N = 30*	*N = 34*				
ASRAP	53.65 (6.04)	52.49 (5.69)	49.31 (8.71)	49.42 (7.81)	–4.38 [–6.35; –2.41]	–3.14 [–4.95; –1.32]	–1.24 [–3.82; 1.33]	0.338

a ANCOVA on comparison between treatments, including pMI imputations for RP attacks end points and RCS.

b For variables not normally distributed statistical analyses were performed on logarithmic values, and change was back transformed. Values are Mean (S.D.) and LS mean [95% CI]. LS: Least Square Mean; RCS: Raynaud’s Condition Score; ANCOVA: analysis of covariance; ASRAP: Assessment of Scleroderma-associated Raynaud’s Phenomenon; NRS: Numerical Rating Scale; DDD: distal dorsal difference; AUC: area under the curve.

### Thermography and cold challenge

There was no statistically significant difference between the vipoglanstat and placebo groups in peripheral blood flow in terms of DDD and mean finger temperature, as assessed by thermography, following the first study drug administration or from baseline to end of treatment (Table 2, Supplementary Table S1, available at *Rheumatology* online). The recovery of peripheral blood flow after the cold challenge did not change following the first administration of the study drug, or from baseline to end of treatment in either the vipoglanstat or the placebo groups (Table 2, Supplementary Table S1, available at *Rheumatology* online).

### ASRAP and global impression of change

**Figure 3.**
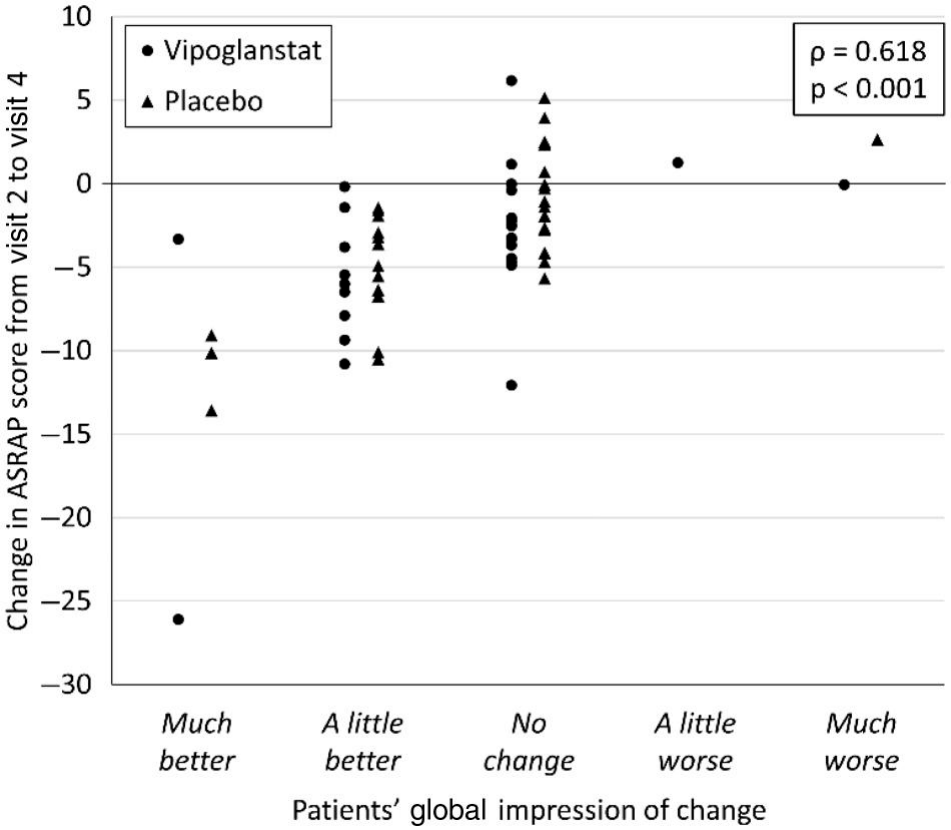
Correlation between Assessment of Scleroderma-associated Raynaud’s Phenomenon (ASRAP) and Patients’ Global Impression of Change (PaGIC). ρ = Spearman’s rho

There was no statistically significant difference between the vipoglanstat and placebo groups in the mean change from baseline to week 4 in the ASRAP scores, which improved in both treatment groups (Table 2). Further, there was no statistically significant difference between the treatment groups for PaGIC or PhGIC, and physicians’ and patients’ ratings were very similar (Supplementary Table S2, available at *Rheumatology* online). *Post hoc* analysis showed high correlation between PaGIC and change in the ASRAP score from baseline to end of treatment (Spearman’s rho = 0.618; *P* < 0.001) (Fig. 3).

### Inhibition of mPGES-1 activity and excretion of arachidonic acid metabolites

**Figure 4.**
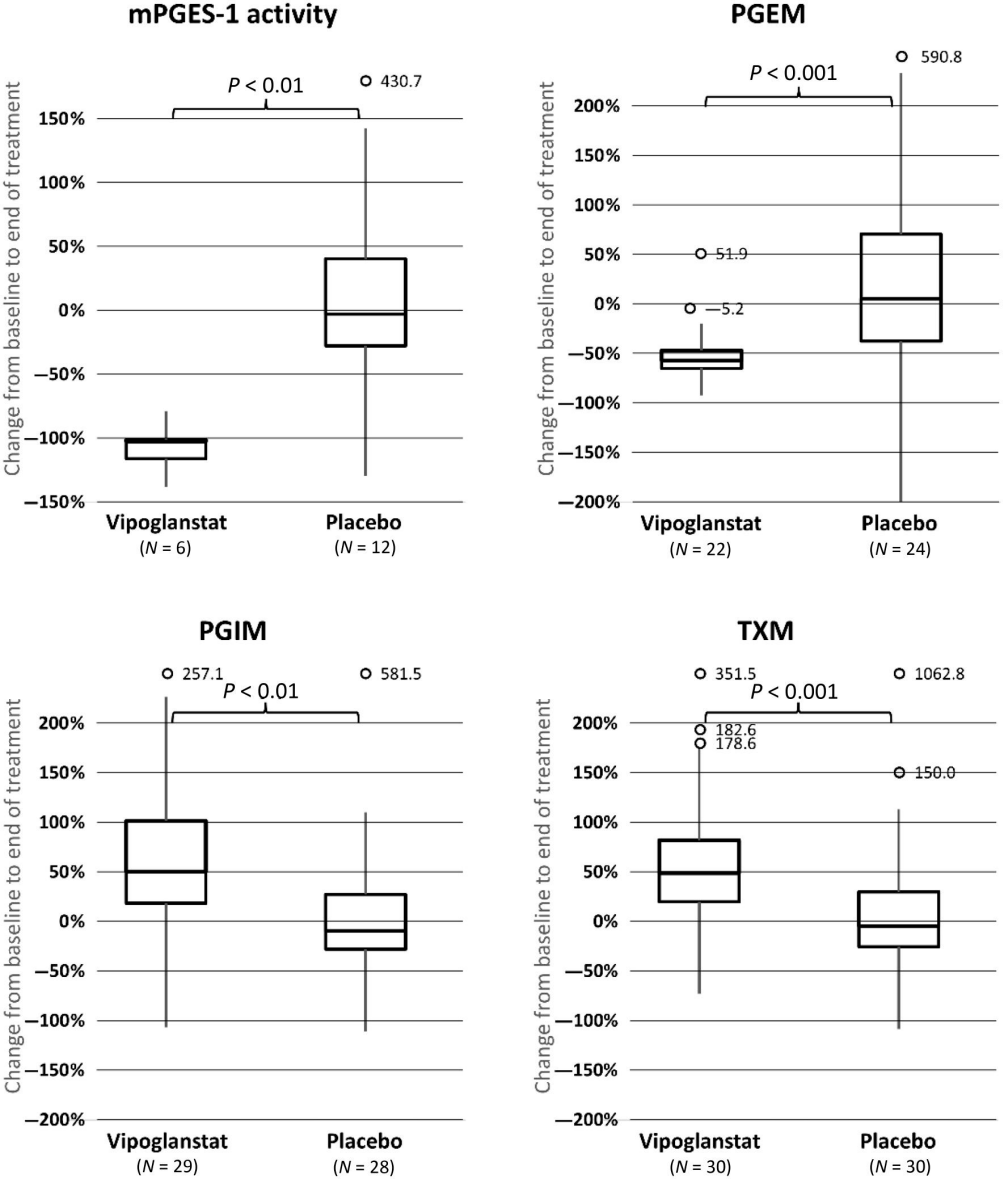
Change from baseline to end of treatment in prostaglandin E synthase-1 (mPGES-1) activity and excretion of arachidonic acid metabolites. mPGES-1 activity was assessed as prostaglandin E_2 (_PGE_2_) synthesis in a whole-blood assay. Urinary excretion of metabolites from PGE_2_ (PGEM), prostacyclin (PGIM) and thromboxane (TXM) were analysed in morning urine before intake of study medication and was normalized to urinary creatinine concentration. For details, see material and methods section. Some samples could not be analysed for technical reasons; thus, the patient was excluded from metabolite analysis if either the visit 2 or visit 4 results were ‘missing’. Box-whisker plot: The boxes indicate the intraquartile range (IQR) and the lines inside the boxes the median values. The whiskers indicate the range of values that are outside of the IQR in a distance ≤1.5 × IQR. Any points outside the whiskers are considered to be outliers and are marked as circles. mPGES-1: prostaglandin E synthase-1; PGEM: metabolite derived from PGE_2_; PGIM: metabolite derived from prostacyclin; TXM: metabolite derived from thromboxane

The median change in mPGES-1–derived PGE_2_ in the whole-blood assay from baseline to ∼24 h after dosing at end of treatment was –102.4% in the vipoglanstat group and –2.8% in the placebo group (*P* < 0.01), demonstrating full inhibition of the enzyme over the full dosing interval (Fig. 4). The median change from baseline to end of treatment in urinary excretion of the PGE_2_ metabolite was –57.2% and +4.9% in the vipoglanstat and placebo groups, respectively (*P* < 0.001). The corresponding values for the prostacyclin metabolite were +49.9% and –9.8% (*P* < 0.01) and for the thromboxane metabolite +48.4% and –5.0% (*P* < 0.001) (Fig. 4).

### Safety

There were no deaths or serious AEs reported in vipoglanstat group. One subject in the placebo group experienced a serious AE, not considered related to treatment or study procedures, classified as a thermal burn (scalding with hot water), leading to hospitalization and complicated with sepsis caused by an unspecified intrahospital bacterium, leading to death.

All patients who had taken at least one dose of the study medication were included in the safety analyses: 33 and 36 patients in the vipoglanstat and placebo groups, respectively. The frequency of AEs was similar in the treatment groups, with 37 AEs in 16 patients (48.5%) in the vipoglanstat group and 45 AEs in 18 patients (50.0%) in the placebo group. AEs classified as possibly or probably related to treatment were reported by 10 (30.3%) patients in the vipoglanstat group and 11 (30.6%) patients in the placebo group. One subject from the vipoglanstat group and two patients from the placebo group experienced AEs that led to discontinuation from the study, and an AE of severe intensity was reported by one subject in each treatment group. The most frequently reported AEs (≥5% of patients in any treatment group) were headache (6 and 4), nausea (2 and 1), upper abdominal pain (2 and 0), arthalgia (0 and 3) and decreased white cell count (0 and 2), in the vipoglanstat and placebo groups, respectively. There were no clinically relevant changes in haematological, blood chemistry or urine analyses. Neither were there any changes in ECG recordings during the study or at the assumed maximal exposure.

## Discussion

This is the first study investigating the effect of an mPGES-1 inhibitor in SSc-related RP. However, despite full inhibition of mPGES-1, leading to decreased systemic biosynthesis of pro-inflammatory PGE_2_ and shunting to increased prostacyclin biosynthesis, no effect on RP-related outcomes or recovery after a cold challenge was observed.

Both the vipoglanstat and the placebo groups improved in RP-related end points, specifically frequency of, duration of and pain during attacks, RCS, ASRAP, PaGIC and PhGIC. This is in accordance with many studies in SSc-related RP [8, 20], and the level of decrease in frequency of RP attacks was similar to the assumptions for the power calculation. This phenomenon has been described as being likely to represent a regression towards the mean [21], but other explanations such as change in behaviour avoiding RP attack triggers during participation in a clinical study cannot be excluded. No improvement was observed in hand and finger temperature or rewarming following a cold challenge; a placebo effect on these assessments is unlikely, since these assessments provide an objective assessment of pathophysiology related to the underlying vasculopathy. The patients studied had well-established disease, with structural vascular abnormalities (as demonstrated by RP-duration and nailfold capillaroscopy findings) and were representative of patients with SSc most in need of effective treatment for RP.

The study provides further confirmation of the feasibility of recording RP attacks using electronic diaries in patients with SSc.

The ASRAP questionnaire is a novel patient-reported outcome (PRO) instrument for assessing the severity and impact of RP in patients with SSc. The instrument was developed by an international consortium of SSc experts and with direct input from patients with SSc throughout the process to achieve the goal of fully capturing the patient experience of SSc-RP [16, 17]. This was the first interventional clinical study including ASRAP as an end point, demonstrating the feasibility of the instrument in that setting. The observed improvement in ASRAP and the high correlation between PaGIC and change in ASRAP score provide useful additional responsiveness data in the validation of this PRO instrument.

Studies on mPGES-1 knock-out mice have demonstrated increases in plasma levels [10] and urinary excretion of prostacyclin metabolites [11]. A shift from the PGE_2_ towards the prostacyclin pathway has also been demonstrated in humans by pharmacological inhibition of mPGES-1 in phase 1 studies with LY3023703 [13] and vipoglanstat [12], where full shunting was already observed during the first day of treatment (Gesynta data on file). The explanation for increased prostacyclin synthesis following mPGES-1 inhibition is a shunting of excess amounts of PGH_2_ to other pathways in the arachidonic acid cascade. The importance of this increased prostacyclin production has been demonstrated in studies on human vessels *in vitro*, which have shown that mPGES-1 inhibitors reduce vasomotor tone under various conditions. Different mPGES-1 inhibitors reduced adrenergic vasoconstriction in resistance arteries obtained from abdominal s.c. fat biopsies, the internal mammary artery, and the saphenous vein [22–24]. Furthermore, acetylcholine-induced dilatation in the resistance arteries increased when exposed to mPGES-1 inhibitors [24].

Vipoglanstat efficiently inhibited mPGES-1, as demonstrated by complete inhibition in whole blood. Systemic PGE_2_ production, measured as urinary PGE_2_ metabolite excretion, was reduced by 57%, indicating that other PGE synthases, important for homeostasis, were less affected (because 43% of PGE_2_ production was still observed). As mentioned above, prostacyclin analogues improve RP symptoms [4, 5] and rewarming after cold challenge [6] in patients with SSc. However, we could not observe any beneficial effects by increasing endogenous prostacyclin synthesis by 50%, as evaluated by urine metabolite excretion. The lack of effect could be due to levels of prostacyclin produced in the digital microvessels being too low. Prostacyclin has a biological half-life of about 1.5 min [25] and is thus active only at, or close to, the site of synthesis. Local levels of prostacyclin cannot be easily measured, and it is possible that the increased synthesis does not take place close enough to the finger microvessels. Although the treatment period was only 4 weeks, because of vipoglanstat’s rapid onset of action any beneficial effect would have been expected to be observed within this time-frame. In conclusion, although vipoglanstat did not confer benefit in SSc-related RP, the study suggested that vipoglanstat was safe and well tolerated in a dose achieving full inhibition of mPGES-1, warranting its further evaluation in diseases where mPGES-1 plays a pathogenetic role.

## Supplementary Material

keae049_Supplementary_Data

## Data Availability

The Sponsor will share de-identified individual participant data collected during the trial with researchers who provide a methodologically sound proposal.
