# Supplementary material for: A phase 2 trial investigating the efficacy and safety of the mPGES-1 inhibitor vipoglanstat in systemic sclerosis-related Raynaud's
Source: Rheumatology (Oxford). 2024 Jan 30;64(2):704–13. doi: 10.1093/rheumatology/keae049 (PMC11781579; doi:10.1093/rheumatology/keae049)
Supplement: keae049_Supplementary_Data [file keae049_supplementary_data.docx]

**Supplement to**

**A phase 2 trial investigating the efficacy and safety of the mPGES-1 inhibitor vipoglanstat in systemic sclerosis-related Raynaud's**

Göran Tornling, Charlotte Edenius, John D. Pauling, Christopher P. Denton, Anna Olsson, Jan Kowalski, Andrea Murray, Marina Anderson, Smita Bhat, Francesco Del Galdo, Frances Hall, Mariusz Korkosz, Dorota Krasowska, Jacek Olas, Vanessa Smith, Jaap Van Laar, Madelon Vonk, Anna Wojteczek, Ariane L Herrick

1. **Eligibility criteria**
2. **Methods for determination of mPGES-1 activity arachidonic acid metabolites in urine**
3. **Study design**
4. **Results**
5. **Eligibility criteria**
   1. **Inclusion criteria**

Individuals who met all of the following criteria were eligible to participate in the study.

1. Subjects must provide signed and dated written informed consent before the conduct of any study-specific procedures.
2. Male and female subjects aged 18-75 years inclusive.
3. Systemic Sclerosis diagnosed according to European League Against Rheumatism (EULAR)/American College of Rheumatology (ACR) criteria[14].
4. Subjects with signs of other autoimmune diseases (e.g. Sjögren’s syndrome, myositis, rheumatoid arthritis) could be included if SSc is the dominating phenotype.
5. Raynaud’s attacks typically ≥7 times per week during the last 4 weeks prior to screening despite background medication (only allowed vasodilatory therapy is calcium channel blockers or PDE-5 inhibitors).
6. Women of childbearing potential (WOCBP) must be using a highly effective method of contraception to avoid pregnancy throughout the study and for 4 weeks after the last dose of IMP in such manner that the risk of pregnancy is minimised.
7. Women must not be pregnant or breastfeeding.
8. Male subjects to agree to use condom in combination with use of contraceptive methods with a failure rate of <1% to prevent pregnancy and drug exposure of a partner, and refrain from donating sperm from the first date of dosing until 3 months after last dosing of the IMP.
9. Ability of subjects to participate fully in all aspects of this clinical trial.
10. In addition to fulfilling all eligibility criteria, subjects must fulfil the following criteria to be randomised:

- ≥7 RP attacks during the last week of the run-in period as captured in the eDiary, with no more than 2 days without RP attacks.
- Compliance with the eDiary during the 7 most recent days prior to baseline (Visit 2), excluding the visit day itself, defined as having submitted ≥5 days of eDiary records (out of a possible 7 days) for RCS and RP during that period.
  1. **Exclusion criteria**

Individuals who met any of the following criteria were not eligible to participate in the study.

1. SSc disease duration of greater than 120 months from first non-Raynaud’s manifestation
2. Current smokers or stopped smoking or used nicotine in any form <3 months prior to Visit 1.
3. Dose-change or initiation of vasodilating substances (calcium blockers or PDE-5 inhibitors) within 4 weeks prior to Visit 1. Subjects are not allowed to use a combination of calcium blockers and PDE-5 inhibitors from 4 weeks prior to Visit 1 and throughout the study.
4. Use of iloprost or other intravenous (iv) or po prostacyclin receptor agonist within 4 weeks prior to Visit 1.
5. Ongoing treatment with immunosuppressive therapies (other than mycophenolate) including, but not restricted to; cyclophosphamide, azathioprine, methotrexate, or cyclosporine, or use of those medications within 4 weeks of trial entry.

*Note: Subjects could be included if they have been treated with a stable dose of mycophenolic acid during 4 weeks prior to Visit 1.*

1. Use of systemic corticosteroids during 4 weeks prior to Visit 1 and during the course of the study.
2. Use of moderate or strong CYP3A4 inhibitors within 5 terminal half-lives or one week, whichever is longer, prior to Visit 2. Examples of a moderate or strong CYP3A4 inhibitors are diltiazem, verapamil, and grapefruit juice.
3. Concurrent serious medical condition, with special attention to cardiovascular conditions, which in the opinion of the investigator makes the subject not suitable for this study.
4. Prolonged QTcF interval defined as a mean QTcF >450 msec at Visit 1, or at Visit 2 (prior randomisation).
5. Creatinine clearance <50 mL/min (determined by Cockcroft-Gault equation) at Screening (Visit 1).
6. Active digital ulcer (DU) within 4 weeks prior to Visit 1.
7. Have known allergies to any components of the vipoglanstat formulation.
8. Clinically meaningful laboratory abnormalities at Screening (Visit 1), as determined and documented by the Investigator.
9. Positive test results for HbsAg, HCVAb or HIV-1 and/or -2 antibodies at Screening (Visit 1).
10. Subjects known or suspected of not being able to comply with this trial protocol (e.g. due to alcoholism, drug dependency or psychological disorder).
11. Subject is mentally or legally incapacitated at the time of screening or has a history of clinically significant psychiatric disorders that would impact the subject’s ability to participate in the study according to the Investigator.
12. Malignancy within the past 5 years except for in situ removal of basal cell carcinoma and cervical intraepithelial neoplasia grade I.
13. Planned major surgery within the duration of the study.
14. Blood donation (or corresponding blood loss) within 12 weeks prior to Visit 1.
15. Participation in another interventional clinical study involving IMP within 4 weeks or given an experimental drug within 5 half-lives, whichever longest, prior to Visit 1.

- 1. **Exclusion criterion for cold challenge**

At Visit 2: Finger temperature below 27°C after acclimatising at an ambient temperature of 23°C (±2°C) for a period of 20 minutes.

1. **Methods for determination of mPGES-1 activity and of arachidonic acid metabolites in urine**
   1. **Determination of mPGES-1 activity**

Whole-blood samples were drawn prior to study drug administration on Visit 2 and Visit 4 and stored at room temperature until the assay was initiated within 45 (± 5) min after collection. The samples were divided in two aliquots; one supplemented with the mPGES-1 inhibitor MF-63 to a final concentration 10 µM to achieve full inhibition of mPGES-1, and the other supplemented with PBS. After 30 minutes at room temperature lipopolysaccharide (LPS) to a final concentration of 30 µg/mL was added to induce expression of mPGES-1, and both aliquots were incubated for 24 hours (37°C, 5% CO_2_). After centrifugation blood plasma was stored at ≤-60°C until PGE_2_ was quantified by LC-MS/MS (Lablytica Life Science AB, Uppsala, Sweden). The mPGES-1 derived PGE_2_ amount in the sample was defined as the difference between the sample incubated with PBS (total PGE_2_) and the sample incubated with MF-63 (non-mPGES-1 derived PGE_2_). The change in mPGES-1 activity from Visit 2 to Visit 4 was defined as the percentage change of mPGES-1 derived PGE_2_.

- 1. **Determination of arachidonic acid metabolites in urine**

Morning urine was collected at Visit 2 and Visit 4 and stored frozen at ≤-60°C. From each urine portion 250 µL was diluted with 10 mM ammonium acetate (pH=9.5) and extracted using mixed mode anion exchange solid-phase extraction. The analytes were eluted in 3% acetic acid in acetone, and dried extracts were redissolved in 50 µL methoxyamine hydrochloride (0.5 mg/mL dissolved in DMF:H_2_O, 1:2) to obtain stable derivatives which were analysed by LC-MS/MS (Lablytica Life Science AB, Uppsala, Sweden). The following metabolites were analysed:

|  | **Metabolite** | **Chemical name** |
| --- | --- | --- |
| PGE_2_ | PGEM  (TetranorPGEM) | 9,15-dioxo-11α-hydroxy-13,14-dihydro-2,3,4,5-tetranor-prostan-1,20-dioic acid or tetranor-prostan-1,20-dioic acid |
| Prostacyclin | PGIM  (2,3-dinor-6-keto-PGF_1_) | 6-oxo-9α,11α,15S-trihydroxy-2,3-dinor-prost-13E-en-1-oic acid, sodium salt or  2, 3-dinor-6-ketoprostaglandin F1α |
| Thromboxane | TXM  (11-dehydro-TXB_2_) | 9α,15S-dihydroxy-11-oxothromba-5Z,13E-dien-1-oic acid or 11-dehydro-thromboxane B_2_ |

The amount of metabolites was normalized by urinary creatinine excretion expressed as (amount metabolite/mg creatinine). The change in excretion of creatinine normalized metabolites from Visit 2 to Visit 4 was defined as the percentage change of the metabolites.

1. **Study design**


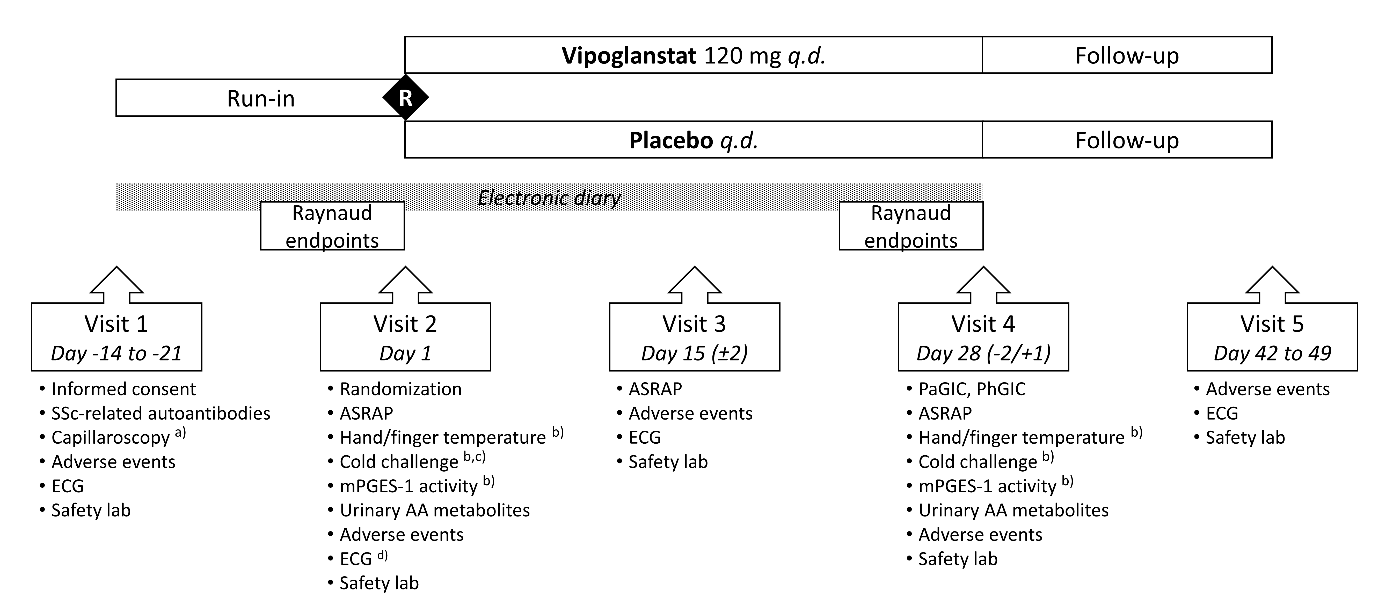


**Figure S1. Study design**

^a^) Could alternatively be performed at Visit 2; ^b)^ Only performed at pre-selected sites; ^c)^ Performed twice, prior to and 180 minutes after study drug administration; ^d)^ Performed twice, prior to and 170 minutes after study drug administration; PaGIC: Patient’s Global Impression of Change; PhGIC: Physician’s Global Impression of Change; ASRAP: Assessment of Scleroderma-associated Raynaud’s Phenomenon questionnaire

1. **Results**

**Table S1. Change from pre to post dose in hand/finger temperature and recovery after cold challenge at baseline**

|  | **Pre dose** | | **Post dose** | | **Change from pre dose to post dose ^a)^** | | | |
| --- | --- | --- | --- | --- | --- | --- | --- | --- |
|  | **Vipoglanstat** | **Placebo** | **Vipoglanstat** | **Placebo** | **Vipoglanstat** | **Placebo** | **Vipoglanstat - Placebo** | **P value** |
| **Hand and finger temperature ^b)^** | N=16 | N=15 | N=16 | N=15 |  |  |  |  |
| DDD (⁰C) | -1.71 (1.47) | -2.43 (1.79) | -1.09 (1.08) | -1.29 (1.31) | 0.83 [0.23; 1.43] | 0.90 [0.27; 1.53] | -0.07 [-0.88; 0.74] | 0.861 |
| Mean finger temperature (⁰C) | 29.15 (2.28) | 28.08 (3.51) | 30.47 (1.90) | 30.25 (2.97) | 1.83 [0.60; 3.05] | 1.78 [0.48; 3.07] | 0.05 [-1.59; 1.69] | 0.951 |
| **Finger temperature after**  **cold challenge** |  |  |  |  |  |  |  |  |
| AUC (⁰C*sec) | 22,670 (2,961) | 22,496 (2,916) | 23,169 (2,774) | 22,543 (2,216) |  |  |  |  |
| ln(AUC) **^c)^** | 10.02 (0.126) | 10.01 (0.121) | 10.04 (0.116) | 10.02 (0.096) | 1.02 [0.98; 1.07] | 1.01 [0.96; 1.06] | 1.02 [0.96; 1.08] | 0.602 |
| Max temperature (⁰C) | 26.8 (4.17) | 26.5 (4.03) | 27.5 (3.83) | 26.7 (3.66) |  |  |  |  |
| ln(Max temperature) **^c)^** | 3.28 (0.151) | 3.27 (0.143) | 3.30 (0.137) | 3.27 (0.133) | 1.03 [0.98; 1.10] | 1.01 [0.96; 1.07] | 1.02 [0.95; 1.10] | 0.600 |

**^a)^** ANCOVA on comparison between treatments including pMI imputations; **^b)^** Assessments were performed after the first cold challenge immediately before and 150 minutes post study drug administration; **^c)^** Variables were not normally distributed and statistical analyses were performed on logarithmic values, and results for change from baseline was presented for back transformed data.

Values are Mean (SD) and LS mean [i.q.r.]

**Table S2. Patient’s and Physician’s Global Impression of Change**

| **Rating of Raynaud’s** | **PaGIC** | | **PhGIC** | |
| --- | --- | --- | --- | --- |
|  | **Vipoglanstat**  N=29 | **Placebo**  N=31 | **Vipoglanstat**  N=30 | **Placebo**  N=34 |
| Much better | 2 (6.9%) | 3 (9.7%) | 1 (3.3%) | 2 (5.9%) |
| A little better | 9 (31.0%) | 12 (38.7%) | 11 (36.7%) | 15 (44.1%) |
| No change | 16 (55.2%) | 15 (48,4%) | 17 (56.7%) | 16 (47.1%) |
| A little worse | 1 (3.4%) | . | . | 1 (2.9%) |
| Much worse | 1 (3.4%) | 1 (3.2%) | 1 (3.3%) | . |

PaGIC: Patients’ Global Impression of Change; PhGIC: Physicians’ Global Impression of Change
